# Use of Social Media to Promote Cancer Screening and Early Diagnosis: Scoping Review

**DOI:** 10.2196/21582

**Published:** 2020-11-09

**Authors:** Ruth Plackett, Aradhna Kaushal, Angelos P Kassianos, Aaron Cross, Douglas Lewins, Jessica Sheringham, Jo Waller, Christian von Wagner

**Affiliations:** 1 Department of Applied Health Research, University College London London United Kingdom; 2 Research Department of Behavioral Science and Health, University College London London United Kingdom; 3 The Policy Research Unit in Cancer Awareness, Screening and Early Diagnosis, Queen Mary University London London United Kingdom; 4 Cancer Prevention Group, School of Cancer and Pharmaceutical Sciences, King’s College London London United Kingdom

**Keywords:** social media, review, cancer, campaign, health promotion, public health, early detection of cancer, cancer screening, health care disparities

## Abstract

**Background:**

Social media is commonly used in public health interventions to promote cancer screening and early diagnosis, as it can rapidly deliver targeted public health messages to large numbers of people. However, there is currently little understanding of the breadth of social media interventions and evaluations, whether they are effective, and how they might improve outcomes.

**Objective:**

This scoping review aimed to map the evidence for social media interventions to improve cancer screening and early diagnosis, including their impact on behavior change and how they facilitate behavior change.

**Methods:**

Five databases and the grey literature were searched to identify qualitative and quantitative evaluations of social media interventions targeting cancer screening and early diagnosis. Two reviewers independently reviewed each abstract. Data extraction was carried out by one author and verified by a second author. Data on engagement was extracted using an adapted version of the key performance indicators and metrics related to social media use in health promotion. Insights, exposure, reach, and differing levels of engagement, including behavior change, were measured. The behavior change technique taxonomy was used to identify how interventions facilitated behavior change.

**Results:**

Of the 23 publications and reports included, the majority (16/23, 70%) evaluated national cancer awareness campaigns (eg, breast cancer awareness month). Most interventions delivered information via Twitter (13/23, 57%), targeted breast cancer (12/23, 52%), and measured exposure, reach, and low- to medium-level user engagement, such as number of likes (9/23, 39%). There were fewer articles about colorectal and lung cancer than about breast and prostate cancer campaigns. One study found that interventions had less reach and engagement from ethnic minority groups. A small number of articles (5/23, 22%) suggested that some types of social media interventions might improve high-level engagement, such as intended and actual uptake of screening. Behavior change techniques, such as providing social support and emphasizing the consequences of cancer, were used to engage users. Many national campaigns delivered fundraising messages rather than actionable health messages.

**Conclusions:**

The limited evidence suggests that social media interventions may improve cancer screening and early diagnosis. Use of evaluation frameworks for social media interventions could help researchers plan more robust evaluations that measure behavior change. We need a greater understanding of who engages with these interventions to know whether social media can be used to reduce some health inequalities in cancer screening and early diagnosis.

**International Registered Report Identifier (IRRID):**

RR2-10.1136/bmjopen-2019-033592

## Introduction

Social media is becoming increasingly popular, estimated to be used by well over half of the world’s population [1,2]. Given social media’s potential for widespread public engagement, it is commonly incorporated into public health interventions [3,4]. The term “intervention” in this article refers to a spectrum of activities ranging from national awareness-raising public health campaigns, such as breast cancer awareness month (BCAM), to more targeted activities that use social media functions like discussion groups to deliver information to specific audiences. Social media interventions can influence behavior by targeting cognitive or emotional responses, generating discussions, and changing social norms [5]. Additionally, social media allows health messages to be disseminated rapidly, at low cost, to large numbers of people across large geographical areas [6-12].

Several national public health campaigns have used social media to try to improve the early diagnosis of cancer through raising awareness of cancer symptoms, encouraging help-seeking, and attempting to influence social norms around help-seeking [13,14]. However, there are concerns that the effects of these campaigns are short-lived, often only involve one-way communication, and do not benefit those in most need [4,5,15,16]. Social media can be used to target messages toward specific geographical regions or demographic groups, such as those who are known to have poor knowledge of cancer symptoms or encounter more barriers to accessing cancer screening [17,18]. Therefore, social media interventions may be more able to address health inequalities than traditional interventions [9,19,20]. However, little is known about the unintended effects of social media interventions and the possibility of spreading misinformation [9,21,22]. Additionally, social media cannot reach those with poor access to digital technology, who may also have the greatest need for public health information [23].

Despite increased use of social media interventions, there is little evidence about whether they improve cancer screening and early diagnosis [24]. There are no review publications that describe the variety of ways that social media specifically is used to promote cancer screening and early diagnosis, how these interventions might facilitate behavior change, and how this has been evaluated. Previous systematic reviews looking at a range of different media-based interventions for cancer screening included very few articles evaluating social networking sites like Facebook and have focused on specific research questions about impact and effectiveness [25,26]. However, use of social media interventions for public health is evolving rapidly and there is a need for a broader mapping of diverse studies to inform future development and evaluations. Therefore, a scoping review methodology was used to map the literature on the ways in which social media has been used to promote cancer screening and early diagnosis and how it was evaluated [27]. Specifically, we aimed to address the following research questions: (1) What are the characteristics of social media interventions that aim to promote cancer screening and early diagnosis?, (2) What are the mechanisms of change by which these interventions promote behavior change?, (3) What methodological approaches have been used to evaluate interventions?, (4) What are the outcomes used to measure the impact of interventions?, and (5) What are the key findings?

## Methods

### Overview

Social media interventions designed to promote cancer awareness and screening were identified using a scoping review guided by the methodology of Arksey and O’Malley [28] and expanded upon by Levac et al [29] and Peters et al [30]. We followed the process outlined in the published protocol and followed the Preferred Reporting Items for Systematic reviews and Meta-Analyses extension for Scoping Reviews (PRISMA-ScR) [31,32].

### Search Strategy

An experienced research librarian helped to develop the search strategy, which included a combination of subject headings and keyword searches. We identified articles by searching five databases: MEDLINE, PsycINFO, Scopus, Web of Science, and CINAHL (Cumulative Index to Nursing and Allied Health Literature). Multimedia Appendix 1 shows the full search strategy used for MEDLINE, which was adapted for the other databases. Additional articles were identified by conducting internet searches for relevant published material and by hand-searching reference lists of included articles. We searched the grey literature for relevant reports not published in peer-reviewed journals. We also contacted the authors of conference abstracts that met inclusion criteria to see if we could include any unpublished results. Organizations and charities related to cancer screening were contacted via email for any relevant published research reports.

### Inclusion and Exclusion Criteria

We considered peer-reviewed articles and non-peer-reviewed reports from the grey literature, as this was a scoping review that aimed to be inclusive and to explore the breadth of relevant research. The findings will help to inform future systematic reviews of the literature that will also assess the quality of the research. Additionally, non–peer-reviewed articles were included because many evaluations of social media interventions are not published in peer-reviewed journals. Social media is also a rapidly evolving area and current insights might not be captured in peer-reviewed literature, where there is typically a lag time between evaluation and publication. We excluded articles and reports not published in English, as we could not feasibly translate the results into English in a valid and reliable way. Articles and reports that discussed social media platforms, including Facebook, Twitter, Instagram, YouTube, Pinterest, and Snapchat, were eligible for inclusion. Articles relating to social media platforms popular in non–English-speaking countries were not excluded, but as a consequence of limiting our search to articles written in English, the focus of this review is on platforms commonly used in English-speaking countries. We included articles published from 2004—as this was the advent of widespread social media use of these platforms—to June 2019 [33]. The reported findings from mass media campaigns were included if social media was the primary focus of the article. We included articles that discussed both qualitative and quantitative methods. We only included articles about interventions that directly targeted cancer screening and early diagnosis, where the primary message of the intervention focused on raising awareness of cancer symptoms, cancer screening, or promotion of help-seeking for potential cancer symptoms. Articles that reported findings solely on the human papillomavirus vaccine were excluded, as wider issues regarding attitudes toward vaccination and misinformation were considered out of the scope of this review. We excluded articles if the participants were cancer patients, survivors, or health professionals. Articles that evaluated static internet pages, such as blog posts, were also excluded from the review.

### Article Screening and Data Extraction

All identified articles underwent two stages of screening: title/abstract screening and full-text screening. Three researchers (APK, AC, and SR) divided the articles and screened them against the inclusion criteria. Each article was independently reviewed by another researcher (AK). If the eligibility of any title/abstract was unclear, it was included in the full-text screening and any discrepancies were reviewed by an additional author and resolved in a consensus meeting. Interrater reliability for title/abstract screening was good (Cohen κ=0.69) [34]. Two researchers (RP and AK) piloted the data extraction approach and three researchers (RP, APK, and AC) completed the data extraction. We extracted data using a Microsoft Access database to collect key information on article characteristics, details of the interventions, methodological approaches, outcome measures, and key findings. A second researcher (RP, APK, or AC) verified the data extractions, and discrepancies were resolved in regular meetings with the entire team.

Table 1 describes the outcomes extracted from the included articles. These were categorized using an adapted version of Neiger et al’s [35] key performance indicators and metrics related to social media use in health promotion, which included insights, exposure, and reach, as well as low-, medium- and high-level engagement. Outcome measures capturing cancer knowledge and intention to attend cancer screening were not accounted for using the original framework but were categorized as high-level engagement, as greater knowledge and intentions are precursors to behavior change [36,37]. We also extracted information on the nature of information that was delivered and shared in interventions by users and developers, such as how many posts were related to health issues and fundraising. Additional data extraction was undertaken by one researcher (RP) to understand the mechanisms by which interventions might promote behavior change by using the behavior change technique (BCT) taxonomy developed by Michie et al [38]. BCTs were identified from the articles but also from campaign websites where possible. Following the scoping review methodology [28-30], we consulted with six people with experience developing and evaluating social media interventions for cancer screening, as well as a cancer patient, to validate our findings [39]. We asked them to comment on our preliminary findings, and their input helped to shape the narrative synthesis of the data.

**Table 1:** Descriptions and examples of outcomes captured during data charting.^a^

Data charting outcome	Description	Examples
Nature of information delivered/shared	Type of messages delivered by intervention or shared by users	Number of messages referring to cancer
Insights	User feedback	Users’ opinions of information
Exposure	Views of social media content	Number of views of posts/tweets
Reach	Interaction with social media content and users’ characteristics	Number of page likes and demographics of users
Low-level engagement	Agreement with the social media content	Number of likes of posts
Medium-level engagement	Users creating or sharing their own social media messages or sharing intervention messages on their own profiles	Number of posts/retweets
High-level engagement (behavior change)	Users’ understanding of the messaging, intention to change their behavior, or actions taken offline related to the desired behavior change	Change in cancer screening attendance

^a^Insights, exposure, reach, and low-, medium-, and high-level engagement were measured using an adapted version of Neiger et al’s [35] key performance indicators and metrics related to social media use in health promotion.

## Results

### Article Selection

**Figure 1:**
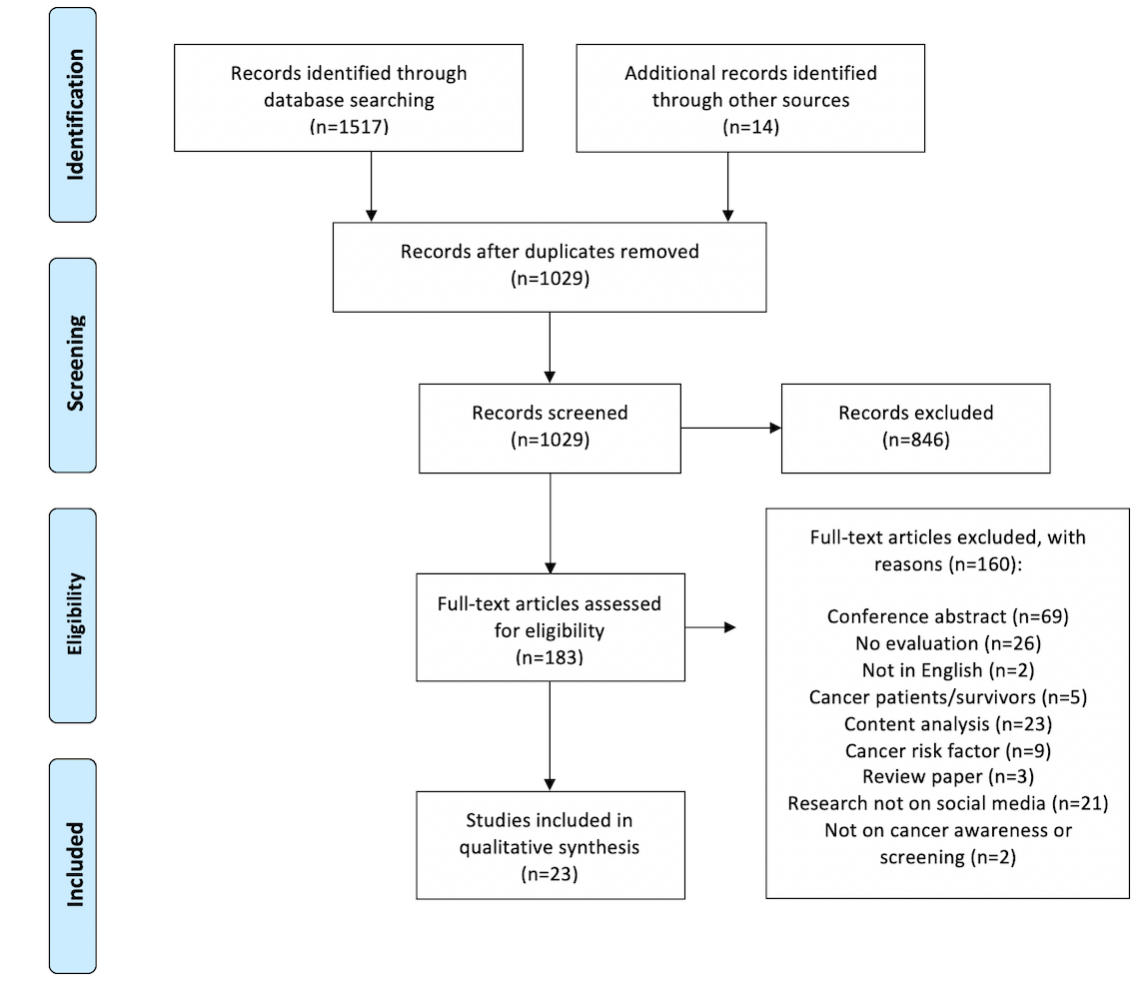
Preferred Reporting Items for Systematic reviews and Meta-Analyses extension for Scoping Reviews (PRISMA-ScR) flow diagram of the literature search and article selection process.

Based on the initial search, 1029 articles were identified after duplicates were removed. We screened a total of 183 full-text articles and included 23 of those articles in this review. Figure 1 outlines the selection process.

### Characteristics of Articles

Multimedia Appendix 2 outlines the characteristics of each included article [40-62]. Only 2 of 23 (9%) articles were from the grey literature and were charity campaign reports; the rest of the articles were published in academic journals. First author institutions represented 10 countries, most commonly the United States (11/23, 48%), Canada (3/23, 13%), and the United Kingdom (2/23, 9%).

### Characteristics of Social Media Interventions

#### Types of Interventions

Multimedia Appendix 2 describes the characteristics of each intervention. We found four different types of social media interventions present in the literature: (1) national cancer awareness month campaigns, (2) regional cancer awareness month campaigns, (3) targeted interventions, and (4) untargeted interventions. Most articles included in this review (16/23, 70%) evaluated national cancer awareness month campaigns, defined as national campaigns to improve cancer awareness and screening delivered to the general public; Multimedia Appendix 3 details an example campaign. Two articles (2/23, 9%) evaluated regional cancer awareness month campaigns. These were campaigns that took place at the same time as national campaigns but were delivered to a specific region [40,41]. Three articles (3/23, 13%) evaluated targeted interventions that used defined activities to deliver cancer awareness and screening information to specific groups and were not part of a cancer awareness month campaign. Multimedia Appendix 4 provides an example of a targeted intervention. Two articles (2/23, 9%) evaluated untargeted interventions that included cancer awareness and screening information delivered to the general public that were not part of an awareness month campaign. One untargeted intervention article (1/23, 4%) explored the impact of a celebrity Tweet about having cancer and the test that saved their life [42]. The other untargeted intervention delivered cancer information videos to the public [43].

#### Cancer Type

Most commonly, the articles reported interventions aimed at breast cancer (12/23, 52%), followed by prostate and testicular cancer (7/23, 30%), cervical cancer (4/23, 17%) [44-47], colorectal cancer (3/23, 13%) [42,48,49], generic cancer (no specific cancer type; 3/23, 13%) [50-52], familial cancer (inherited tumors; 1/23, 4%), and lung cancer (1/23, 4%) [42,43]. Some articles evaluated interventions for more than one cancer type (7/23, 30%).

#### Intervention Source

Interventions were most commonly delivered by cancer charities (17/23, 74%), followed by public health government bodies (4/23, 17%) [40,44,47,53], regional health services (2/23, 9%) [43,54], a university (1/23, 4%) [43], and a celebrity (1/23, 4%) [42].

#### Social Media Platform

Interventions were most commonly delivered via Twitter (13/23, 57%), followed by Facebook (8/23, 35%), YouTube (3/23, 13%) [44,49,51], Instagram (2/23, 9%) [49,52], and Snapchat (1/23, 4%) [54]. Some articles evaluated interventions delivered via more than one platform (4/23, 17%).

#### Nature of Cancer Information Delivered and Shared in Interventions

Just over one-half of the articles (13/23, 57%) analyzed the messages that were delivered and shared by users and intervention developers. Several articles reported that most posts for national campaigns contained non-health messages and nonactionable messages [46,48,51,55-59]. During the 2013 Canadian Movember campaign, there were significantly more tweets on non-health topics, such as moustache growing (n=3549), than on health topics (n=673); only 0.6% (25/4222) of tweets analyzed were about cancer [56]. Furthermore, national campaigns heavily promoted online purchasing and fundraising to support cancer charities [55,57]. Bravo and Hoffman-Goetz [57] found that posts about fundraising and purchasing often did not mention cancer; only 2% (18/819) of fundraising tweets identified prostate or testicular cancer as the reason why they were fundraising. Additionally, for breast, cervical, prostate, and testicular cancer, gendered imagery and language were used to engage users [45,55,57]. In the Movember campaign, 9% (204/2400) of tweets analyzed used war metaphors, with users describing themselves as an “army,” and the moustaches characterized as being “manly” [57].

### Mechanisms of Behavior Change

#### BCTs Used in Interventions

No articles reported theories that informed the development of the intervention. Table 2 details the 10 BCTs that we tentatively identified as being present in the interventions [38]. All interventions targeted cancer screening behaviors such as improving attendance at cancer screening. This was achieved by providing information on cancer screening but also by raising awareness via fundraising activities.

**Table 2:** The number of interventions that used possible behavior change techniques (n=23) [38].

Behavior change techniques used in interventions	n (%)
Credible sources (eg, health professional, government, charity, celebrity)	23 (100)
Information about health consequences	23 (100)
Instruction on how to perform a behavior	22 (96)
Social support	17 (74)
Social comparison (eg, comparing a person’s actions to the actions of others)	17 (74)
Information about others’ approval	17 (74)
Goal-setting behavior	16 (70)
Social incentive (eg, providing a written reward only if a person performs the desired action)	12 (52)
Salience of consequences (eg, emphasizing the consequences of cancer)	2 (9)
Restructuring the physical environment (ie, changing the environment to facilitate the desired action)	1 (4)

The main way in which interventions appeared to influence behavior change was by providing information about the health consequences of cancer, often by providing links to cancer charity/public health websites to access cancer symptom and screening information [55,60]. The information delivered by interventions was all from credible sources, such as government bodies or cancer charities, but information from users may have been less credible. Information was made salient to users in posts that mentioned how users and members of their family were at risk of cancer (eg, “your children and family depend on it”) [55,60]. Approximately three-quarters (17/23, 74%) of the articles reported interventions that used social support, social comparisons, and others’ approval to encourage cancer screening. This was demonstrated by the sharing of personal stories and messages of support from the public, charities, organizations, cancer survivors, and celebrities [46,55,57]. One article reported an intervention that restructured the environment by improving access to booking breast screening appointments through Facebook Messenger [61]. Interventions also influenced behavior change through goal setting and providing incentives. For example, many campaigns encouraged users to complete fundraising goals, such as growing a moustache for Movember to raise awareness [55-58]. They used social incentives like congratulating and thanking users on social media for taking part in fundraising activities to promote cancer screening [50,60].

### Methodological Approaches Used to Evaluate Interventions

Multimedia Appendix 5 provides a breakdown of the methods used in each article. In most of the included articles (20/23, 87%), quantitative research designs were used; one-quarter (5/20, 25%) of those were experimental research designs. A single-group posttest design was the most commonly used experimental design (3/5, 60%) [40,43,44]. Two articles (2/5, 40%) that used experimental designs used two-group pre- and posttest research designs [47,54]. Most (15/20, 75%) of the quantitative articles used observational research designs, where the researchers observed the impact of previously developed interventions. Just over one-half (8/15, 53%) of these were longitudinal studies. One-half (4/8, 50%) of the longitudinal studies measured outcomes over a period of 1 to 2 years [50-53]. Two (2/23, 8.7%) articles used qualitative research designs [55,57] and thematically analyzed social media messages. One (1/23, 4%) article used mixed methods by conducting a single-group pre- and posttest design and thematic analysis of social media messages [61].

### Outcomes Used

Multimedia Appendix 5 shows the outcomes measured for each included article. Most (18/23, 78%) articles measured at least one of Neiger et al’s [35] key performance indicators related to social media use in health promotion. Two (2/23, 9%) articles assessed insights by measuring user opinions [40,43]. Six (6/23, 26%) articles measured exposure, the most common indicator being impressions (the number of times a post was viewed; 4/6, 67%) [40,41,49,62]. Three (3/23, 13%) articles measured reach and the demographics of followers [40,49,52]. Five (5/23, 22%) articles measured low-level engagement by measuring the number of likes on a post [40,46,49,50,53]. Many (11/23, 48%) of the included articles measured medium-level engagement, with the most common indicator being the number of posts/tweets by users (8/11, 73%) [42,45,48,51,52,59,62]. Five articles (5/23, 22%) measured indicators of high-level engagement. Two (2/5, 40%) of these articles measured knowledge of cancer symptoms and screening [47,54] and one (1/5, 20%) article measured intention to get screened [40]. One (1/5, 20%) article measured participation in an offline advocacy event as a volunteer [41] and one (1/5, 20%) measured the number of people who attended screening [61].

### Key Article Findings

#### Insights, Exposure, and Reach

Multimedia Appendix 5 presents the findings of each included article. Exposure varied by the type of intervention that was being evaluated. For example, one evaluation of a national campaign reported over 2 million Facebook impressions [49], and a regional campaign had 53,317 Facebook impressions [40]. Two (2/23, 9%) articles reported user insights and found social media was an acceptable way to deliver cancer awareness and screening information [40,43]. One (1/23, 4%) article found that 88% (43/49) of women surveyed indicated that they were neutral or agreed with seeing mammogram information on Facebook [40].

Three (3/23, 13%) articles suggested that reach varied by gender but only discussed the reach to women and men and no other gender identities. Content on YouTube may have reached more men than women, but Facebook content may have reached more women than men [40,43,49]. One (1/23, 4%) article found Facebook content had a wider reach to those aged 45 to 64 years than to other age groups [40]. Another (1/23, 4%) article suggested that campaigns tended to reach more White users (93%) than African American (7%) or Asian or Hispanic users (0.6%) [49].

#### Low-Level and Medium-Level Engagement

One (1/23, 4%) article showed that users most commonly interacted with campaigns on Facebook by liking posts, followed by sharing content and commenting [50]. Two (2/23, 9%) articles showed that social media influencers and celebrities increased the number of likes due to their large number of followers [46,49]. Three (3/23, 13%) articles found that retweeting was significantly more likely if the tweet was posted by celebrities, organizations, someone with a high number of followers, or someone who frequently tweeted about the campaign [42,46,62]. Three (3/23, 13%) articles reported that posts with images were the most liked and were more likely to be retweeted than posts with just text [50,53,59].

Five (5/23, 22%) articles reported that engagement increased during the campaigns and decreased to baseline levels or below after the campaigns [41,50-52,62]. Two (2/23, 9%) articles found that health information–sharing tweets about cancer tended to rise during campaigns [42,48]. Two (2/23, 9%) articles reported that breast cancer campaigns had much more traffic on social media than other cancer campaigns, even on months that were dedicated to raising awareness of other cancers [42,51]. For example, even though the campaigns for prostate cancer awareness occurred in November, breast cancer received more mentions on Twitter in November than prostate cancer (284,015 posts versus 65,820 posts, respectively) [51]. Two (2/23, 9%) articles reported that colorectal cancer received the least attention on social media compared with breast, prostate, and cervical cancer [42,52]. Engagement with campaigns may vary by ethnicity, as one (1/23, 4%) article found White users consistently mentioned breast and prostate cancer more than other ethnicities [52].

#### High-Level Engagement

One (1/23, 4%) article reported that 9000 participants took part in an offline advocacy event that was part of a regional social media BCAM campaign [41]. Two (2/23, 9%) articles reported that targeted interventions improved knowledge of cancer symptoms and screening compared with a control [47,54]. One (1/23, 4%) article found that a regional mammography campaign improved intention to attend cancer screening; 82% of 49 women surveyed expressed an intent to get a mammogram in the next year [40]. One (1/23, 4%) targeted intervention reported an increase in the number of people who attended a breast screening appointment; attendance increased by an average of 12.9% across seven screening sites in North Midlands, United Kingdom (see Multimedia Appendix 4) [61].

## Discussion

### Principal Findings

Most studies of social media interventions have evaluated national cancer awareness month campaigns, using observational studies to measure exposure, reach, and low- to medium-level engagement with a campaign. A small number of studies suggested that regional cancer awareness month campaigns and targeted interventions might improve cancer awareness, as well as screening intentions and uptake. There was evidence that exposure, reach, and engagement with the interventions varied by age, gender, and ethnicity of users, and also by cancer type.

This scoping review was the first to focus on social media evaluations of interventions to improve cancer screening and early diagnosis. It added to the literature by highlighting the limited number of robust evaluations that captured high-level engagement/behavior change, such as attendance at cancer screening. Evaluating high-level engagement is challenging because timely observational data on cancer screening attendance can be difficult to access and link with social media data [24]. Evaluation is also challenging because social media interventions are often designed without evaluation in mind [3]. During the consultation for this review, experts commented that campaigns are set up so quickly that there is not always time to consider evaluation. Improving cancer screening and early diagnosis is seen as a long-term goal that will take many years to realize, so immediate changes might not be expected or measured. A comprehensive evaluation framework that incorporates elements from behavior change theories and social media engagement frameworks could foster more robust evaluations that capture outcomes that demonstrate impact on behavior change and engagement [17,63,64]. However, as noted during the consultation for this review, there are further challenges to evaluation, such as the difficulty of demonstrating that a specific campaign caused a change in outcomes, as well as the limited time and resources of organizations to conduct evaluations.

This review was the first to use the BCT taxonomy to identify a variety of BCTs that social media interventions used to change health behaviors, including social support and providing information about health consequences [38]. One article reported restructuring the environment to provide better access to cancer screening [61]. As noted during our consultation, governance and data protection issues often limit the ability of health providers to use social media to improve access to care. These issues need to be addressed if interventions are to be tested or implemented on a larger scale. Many articles also reported that information delivered and shared during national cancer awareness month campaigns consisted of more non-health messages relating to fundraising than health messages relating to cancer. For many of these campaigns, the theory of change may be that fundraising messages increase cancer awareness, thereby increasing help-seeking or uptake of screening. Behavior change theory suggests that providing actionable health messages, such as information on cancer symptoms, could influence behavior change more directly than fundraising messages [12,38,63,65,66]. Future evaluations are needed to test our assumptions about how national campaigns might lead to behavior change and what messages would be most effective.

We identified a need for more social media interventions targeted toward colorectal and lung cancer, as most of the articles in this review were evaluations of social media interventions for breast and prostate cancer awareness and screening. Survival rates for lung and colorectal cancer are poorer than for breast and prostate cancer, which is partly because of poor uptake of cancer screening and delayed help-seeking that can lead to a delayed diagnosis [67]. Previous studies [68,69] have shown that there is more stigma around lung cancer than other cancers, and higher perceptions of cancer stigma are associated with delays in seeking medical care. Social media interventions could play a key role in changing social norms and stigma around help-seeking and screening for these cancers [70]. BCTs used by current breast cancer campaigns, such as sharing personal stories, could help to create social support and influence how people view these cancers, which in turn could encourage help-seeking behavior, increase screening uptake, and improve health outcomes.

This review also added to the literature by exploring to what extent inequalities in cancer screening and early diagnosis were measured and potentially addressed by social media interventions. We found some evidence that social media interventions have poorer reach and engagement with ethnic minorities, but there was no information on engagement with other minority groups [52]. Individuals from ethnic minorities might have less interaction with social media campaigns and not seek out cancer screening information because they have less access to cancer screening and higher cancer stigma [68,71-74]. The written and visual communication in social media interventions may also exclude ethnic minorities if the information is only available in English, presented in inaccessible language, or framed in a way that is unrelatable [74]. A lack of social media influencers or campaign role models that resonate with ethnic minorities may also make it less likely that they undertake a behavior, as suggested by social cognitive theory [75]. Future evaluations of social media interventions should measure inequalities in exposure, reach, and engagement and consider their success directly in relation to the groups that they were seeking to target. Targeting interventions toward those with a disproportionate disease burden could help to improve health inequalities seen in cancer screening and early diagnosis. However, social media interventions will have little impact on those who do not use social media, who may also be those in greatest need of information on cancer screening.

Some differences in the use of social media platforms by gender and age were found in this review [49,62]. Facebook content reached more women than men, and reached older-aged adults, and YouTube content reached more men than women [40,43,49]. This is consistent with recent data on social media use in the United Kingdom by age and gender [76]. This highlights the importance of identifying which platforms target users are currently more likely to engage with when designing social media interventions. However, as noted during our consultation, social media is a constantly changing landscape, so messaging needs to be continually updated and transferred to different platforms. Targeting messages also has potential risks, such as the threat to privacy and ethical issues, and it often requires payment and significant time and resources [17,24]. Additionally, data may not be available for particular target users; for example, we only found evidence to show how best to target those who identify as male and female. Previous research has shown that there are gender identity disparities in cancer screening, and trans and nonbinary individuals could benefit from more information regarding cancer screening and early diagnosis [77,78]. Further research is needed to understand the most effective way of targeting social media interventions toward these individuals.

### Limitations

We acknowledge that scoping reviews have several limitations, but a scoping review allowed us to gain a wide-ranging understanding of the role of social media in cancer screening. Research into social media is rapidly growing and this scoping review is a snapshot of evidence for social media interventions at a particular time [26]. Furthermore, as we only selected studies written in English that did not include information about some popular social networking sites in non–English-speaking countries, the findings of this review might not be generalizable. Many of the social media campaigns in this review were also part of multimedia campaigns. Therefore, it is difficult to know whether changes in engagement with social media or health behaviors were due to the social media element of those campaigns and how social media interacts with other aspects of the campaign. Additionally, the coding of BCTs in this review was dependent on reported content and online sources, so there was insufficient detail to identify all techniques used. The review was also dependent on what outcomes the evaluations chose to measure and report. There is currently no protocol for how to report evaluations of social media interventions, so there may be some degree of reporting bias in included articles. Future research would benefit from the development of a reporting protocol based on current frameworks for evaluating social media research [35].

### Conclusions

This review found that most evaluations of social media interventions to improve cancer screening and early diagnosis did not report behavior change outcomes. The limited available evidence suggests some types of social media interventions may improve cancer awareness and intended and actual uptake of screening. Use of evaluation frameworks and reporting guidelines could help future researchers to plan robust evaluations of social media interventions that capture outcomes of behavior change and explore how these interventions work. Future evaluations could also measure who engaged with these interventions to assess whether social media interventions for cancer screening and early diagnosis can address some health inequalities. Interventions focusing on cancers that have received less social media attention, such as colorectal and lung cancer, could help to influence social norms around help-seeking and screening uptake for these cancers, which could improve health outcomes for patients.

## Appendix

Multimedia Appendix 1 MEDLINE search strategy.

Multimedia Appendix 2 Characteristics of included articles and interventions ordered by social media platform and target cancer of interventions.

Multimedia Appendix 3 Description of the Movember campaign.

Multimedia Appendix 4 Description of a targeted media intervention using Facebook.

Multimedia Appendix 5 Characteristics of the evaluations ordered by research design, including sampling, duration, outcome, and key findings.

## Abbreviations

BCAM: breast cancer awareness month
BCT: behavior change technique
CINAHL: Cumulative Index to Nursing and Allied Health Literature
PRISMA-ScR: Preferred Reporting Items for Systematic reviews and Meta-Analyses extension for Scoping Reviews
