# Supplementary material for: Use of Social Media to Promote Cancer Screening and Early Diagnosis: Scoping Review
Source: J Med Internet Res. 2020 Nov 9;22(11):e21582. doi: 10.2196/21582 (PMC7683249; doi:10.2196/21582)
Supplement: Multimedia Appendix 1 [file jmir_v22i11e21582_app1.docx]

## Multimedia Appendix 1

### Search strategy for MEDLINE

MEDLINE (OVID) and Epub Ahead of Print, In-Process & Other Non-Indexed Citations and Daily <1946 to June 06, 2019>

--------------------------------------------------------------------------------

1 Social Media/ (5759)

2 online social networking/ (30)

3 "web 2.0".mp. (624)

4 facebook.mp. (2868)

5 twitter.mp. (2415)

6 instagram.mp. (282)

7 pinterest.mp. (49)

8 snapchat.mp. (41)

9 youtube.mp. (1448)

10 1 or 2 or 3 or 4 or 5 or 6 or 7 or 8 or 9 (9959)

11 exp *Health Knowledge, Attitudes, Practice/ (56098)

12 exp *Neoplasms/ (2761991)

13 11 and 12 (5060)

14 early diagnosis/ or "early detection of cancer"/ (44870)

15 ((cancer* or tumo?r* or malignan* or neoplasm*) adj3 (awareness* or appraisal* or knowledge or informed or recogn*)).mp. (20631)

16 ((cancer* or tumo?r * or malignan* or neoplasm*) adj3 (symptom* or "red flag")).mp. (13786)

17 ((cancer* or tumo?r * or malignan* or neoplasm*) adj3 screen*).mp. (46196)

18 ((cancer* or tumo?r * or malignan* or neoplasm*) adj3 (diagnos?s* or stag?* or detect*)).mp. (384121)

19 13 or 14 or 15 or 16 or 17 or 18 (460408)

20 10 and 19 (94)

21 limit 20 to english language (94)

***************************
