# Supplementary material for: Use of Social Media to Promote Cancer Screening and Early Diagnosis: Scoping Review
Source: J Med Internet Res. 2020 Nov 9;22(11):e21582. doi: 10.2196/21582 (PMC7683249; doi:10.2196/21582)
Supplement: Multimedia Appendix 2 [file jmir_v22i11e21582_app2.docx]

| First author (year), [ref] | Target cancer(s)^a^ | Intervention | Intervention type | Intervention target users | Intervention target location | | Intervention source |
| --- | --- | --- | --- | --- | --- | --- | --- |
| Twitter |  |  |  |  |  | |  |
| Chung (2017) [59] | Breast | BCAM^b^ | National^c^ | Public | US | | Charity |
| Diddi (2017) [60] | Breast | BCAM | National | Public | US | | Charity |
| Thackeray (2013) [62] | Breast | BCAM | National | Public | US and UK | | Charity |
| Bravo (2016) [56] | Prostate and testicular | Movember | National | Public | Canada | | Charity |
| Bravo (2016) [57] | Prostate and testicular | Movember | National | Public | Canada | | Charity |
| Bravo (2017) [58] | Prostate and testicular | Movember | National | Public | Canada, US, and UK | | Charity |
| Lenoir (2017) [45] | Cervical | European cervical cancer prevention week | National | Public | Europe | | Charity |
| Teoh (2018) [46] | Cervical | Cervical cancer awareness month | National | Public | US and UK | | Charity |
| Lee (2016) [48] | Colorectal | Colorectal cancer awareness month | National | Public | South Korea | | Charity |
| Vos (2019) [42] | Prostate, breast, colon, and lung | Response to celebrity announcement of cancer | Untargeted^d^ | Public | US | | Celebrity |
| Facebook |  |  |  |  |  | |  |
| Abramson (2015) [55] | Breast | BCAM | National | Public | US | | Charity |
| Good Things Foundation (2018) [61] | Breast | Information on breast screening and booking appointments via Facebook | Targeted^e^ | Women over age 50 | North Midlands, UK | | Health service and charity |
| Klippert (2018) [40] | Breast | Mammography campaign | Regional^f^ | Men and women over age 40 | 9 counties in Idaho, US | | Government body |
| Theiss (2016) [53] | Breast | Public education campaigns— Know: BRCA (breast cancer gene) and Bring Your Brave | National | Public | US | | Government body |
| Lai (2015) [47] | Cervical | Information on cervical cancer and discussion groups | Targeted | Women; students aged 15-17 years | Taiwan | | Government body |
| Fernández-Gómez (2016) [50] | Prostate and generic | World Cancer Day and Day Against Prostate Cancer | National | Public | Argentina, Colombia, Chile and Spain | | Charity |
| YouTube |  |  |  |  |  | |  |
| Cooper (2016) [44] | Cervical | Gynecologic cancer information videos | National | Women | US | | Government body |
| Jones (2016) [43] | Familial | Familial cancer information videos | Untargeted | Public | Worldwide | | Health service and university |
| Snapchat |  |  |  |  |  | |  |
| Alanzi (2018) [54] | Breast | Breast cancer awareness information | Targeted | Women; university students | Dammam region, Saudi Arabia | | Health service and charity |
| Multiple platforms | |  |  |  |  |  | |
| Vraga (2018)^g^ [51] | Breast, prostate, and generic | World Cancer Day, BCAM, and Movember | National | Public | US | | Charity |
| Xu (2016)^h^ [52] | Breast, prostate, and generic | World Cancer Day, BCAM, and Movember | National | Public | US | | Charity |
| Salako (2017)^I^ [41] | Breast | Part of BCAM | Regional | Public | Lagos State, Nigeria | | Charity |
| Digestive Cancers Europe (2019)^j^ [49] | Colorectal | European colorectal cancer and screening | National | Public | France, Spain, Portugal, Italy, Finland, and Slovakia | | Charity |

^a^Colors represent the type of cancer that the intervention targeted: pink=breast cancer, blue=prostate and testicular cancer, purple=cervical cancer, orange=colorectal cancer, green=familial cancer, and grey=multiple cancers.

^b^BCAM: breast cancer awareness month.

^c^National: national awareness month campaign.

^d^Untargeted: cancer information delivered to general public and not an awareness month campaign.

^e^Targeted: defined combination of activities delivered to a specific group and not an awareness month campaign.

^f^Regional: regional awareness month campaign.

^g^Twitter and Instagram.

^h^Twitter and Instagram.

^i^Twitter and Facebook.

^j^Facebook, YouTube, and Instagram.
