# Supplementary material for: Use of Social Media to Promote Cancer Screening and Early Diagnosis: Scoping Review
Source: J Med Internet Res. 2020 Nov 9;22(11):e21582. doi: 10.2196/21582 (PMC7683249; doi:10.2196/21582)
Supplement: Multimedia Appendix 3 [file jmir_v22i11e21582_app3.docx]

Multimedia Appendix 3: Description of the Movember campaign

| **Movember campaign (2013), Canada [51-53]**  Movember is an annual campaign which aims to create “conversations about men’s health that lead to awareness and understanding of the health risks men face”, including prostate and testicular cancer. A key aspect of the campaign is to encourage men to grow moustaches in November.  Researchers aimed to find out if the campaign goals were achieved by analyzing a random sample of tweets about the November 2013 campaign. This comprised thematic and content analysis of social media quotes, categorization of tweets as health-related or non-health related, and comparison across countries (UK, USA and Canada).  They found that most tweets were non-health related i.e. not directly referencing prostate and testicular cancer and very few tweets contained actionable health information (<1%). Most tweets were about fundraising and moustache growing. They concluded that the campaign did not meet the goal of creating conversations about men’s health issues. |
| --- |
