# Supplementary material for: Use of Social Media to Promote Cancer Screening and Early Diagnosis: Scoping Review
Source: J Med Internet Res. 2020 Nov 9;22(11):e21582. doi: 10.2196/21582 (PMC7683249; doi:10.2196/21582)
Supplement: Multimedia Appendix 4 [file jmir_v22i11e21582_app4.docx]

Multimedia Appendix 4: Description of a targeted media intervention using Facebook

| **North Midlands Breast Screening Facebook Page (2018) [54]**  In 2017-2018, The North Midlands Breast Screening Service set up a Facebook page run by health improvement practitioners to provide information on breast screening and simplify the booking process. Practitioners posted information about screening and responded to questions posted or private messages. A key aspect of this intervention was the ability to book appointments directly through the messaging function.  In addition to peer-to-peer sharing on Facebook, sponsored posts were used to advertise the page to the target group. The service advertised their page through local community groups on Facebook who may have had barriers to accessing screening.  Good Things Foundation compared attendance at breast screening over years and found a 13% increase across 7 sites (range 9%-26%). Qualitative analysis of posts found that the service was a timely, easy and social way to engage with services. |
| --- |
