# Supplementary material for: Use of Social Media to Promote Cancer Screening and Early Diagnosis: Scoping Review
Source: J Med Internet Res. 2020 Nov 9;22(11):e21582. doi: 10.2196/21582 (PMC7683249; doi:10.2196/21582)
Supplement: Multimedia Appendix 5 [file jmir_v22i11e21582_app5.docx]

Multimedia Appendix 5: Characteristics of the evaluations ordered by research design, including sampling, duration, outcome, and key findings

| **First author (year)** | **Sample** | **Duration of intervention evaluated** | **Outcomes^[[1]](#footnote-1)^** | **Key findings** |
| --- | --- | --- | --- | --- |
| Quantitative – experimental research designs | | | | |
| Non-randomized, two group pre and post-test | | | | |
| Alanzi (2018) | Intervention group n= 100 Control group n= 100. Females, university students | 4 weeks | High-level engagement | Intervention significantly improved breast cancer awareness compared to controls who received no intervention (mean 14.8 vs 5.1 on a scale of 0-18). |
| Lai (2015) | Intervention group n= 100 Control group n= 100. 15-17 years old | September-December 2012 | High-level engagement | Intervention group’s knowledge of cervical cancer improved significantly compared to control group (mean 15.1 vs 12.4, on a scale of 0-20). |
| Single group post-test design | | | | |
| Cooper (2016) | US public | July 2012- November 2013 | Exposure | Keyword-targeted videos were viewed more times and were more likely to be watched to completion than pre-roll videos^[[2]](#footnote-2)^ (15.3 million views vs 9.2 million and 44.4% played to completion vs 17.0%). |
| Jones (2016) | General public | 2011-2015 | Insights, exposure, reach | 7/8 found the videos useful. Men were more likely to view videos on YouTube than women. The average age of the registered user was 45–54 years. |
| Klippert (2018) | Idaho, US public (plus 49 Idaho women who completed survey) | October 2017 | Nature of information, insights, exposure, reach, low-level, medium-level and high-level engagement | The cost of screening was seen as a barrier to participation. 88% of women indicated they were comfortable seeing more mammogram information on Facebook. Posts reached 48,503 users. More viewers were women (36,583) than men (10,733). Viewers were mostly aged 45–64. 1,189 campaign likes, 43 comments and posts were shared 204 times. 82% of 49 women who completed the survey intended to get a mammogram in the next year. |
| Quantitative – observational research designs | | | | |
| Cross-sectional | | | | |
| Bravo (2016b) | 4222 tweets | November 2013 | Nature of information | There were significantly fewer health-related (n=673) than non-health-related (n=3549) tweets. Few tweets (0.6% of all tweets) referenced prostate or testicular cancers. Community engagement activities and moustache and grooming references were the most frequent topics in the health-related (10.5 and 2.0%) and non-health-related (32.8 and 32.8%) categories. |
| Bravo (2017) | 12,666 tweets | November 2013 | Nature of information | Few tweets (n=84, 0.7% of 12,666 tweets) provided actionable health information. Moustache was the most popular topic in US tweets, conversations about community engagement were most common in Canadian and UK tweets. |
| Chung (2017) | 1018 tweets | October 2014 | Nature of information, Medium-level engagement | Tweets were about: raising awareness (18.2%), raising monetary support (17.1%), promoting sales activities (16.7%). 6.9% were to call for action e.g. “Get screened”. Retweeting was significantly higher if: posted by an organization than individuals; poster had more followers; poster frequently tweeted about campaign and included photos. |
| Diddi (2017) | 2961 tweets | October 2014 | Nature of information | Most prevalent Health Behavior Model construct in tweets were “perceived barriers” (n=764, 26%) “cues to action” (n=746, 25%). Most tweets posted by breast cancer social media (n=2365, 80%), then nonprofit organization (n=280, 10%), news outlet (n=180, 6%), and a hospital (n=136, 5%). |
| Digestive Cancers Europe (2019) | Over 2 million Facebook impressions | March 2019 | Exposure, reach, Low-level engagement | Over 2 million Facebook impressions. Clickthrough rate was above 1.4%. Women were more likely to watch videos. YouTube clickthrough rate=0.6%. Shorter videos average clickthrough rate=1.6% vs 0.8% for longer videos. Two posts by social influencers secured over 170,000 followers. Post by social influencer received 3755 likes. |
| Lenoir (2017) | 1881 tweets | January 2015 | Nature of information, reach, medium-level engagement | 32.3% of tweets were sensitizing (awareness-raising). Factors associated with posting sensitizing tweets were women who experienced an abnormal smear test, females, living in the UK. 57.4% of users were women. 54.4% of posts were people sharing images of themselves with smeared lipstick. |
| Teoh (2018) | 348 most popular (top) tweets | January 2015 | Nature of information, reach, low-level and medium-level engagement | Health organizations produced 21% of tweets, healthcare professionals 4% and celebrities 4%. 70% of tweets focused on screening. 305 tweets (89%) were fact-based. The median number of followers was 6,384. Median number of retweets =5, median number of likes =4. A celebrity tweet generated >1000 likes and retweets. |
| Longitudinal | | | | |
| Fernández-Gómez (2016) | 484 Facebook messages | November 2015-February 2016 | Nature of information, exposure, low-level and medium-level engagement | 32% of the messages served as support and assistance to the followers (160 messages). Users most commonly interacted by liking posts (251,740), followed by sharing content (88,376) and commenting (4551). |
| Lee (2016) | 10,387 tweets | August-September 2014 | Nature of information, Medium-level engagement | 57.8% of users were individuals, 5.8% were organizations/communities; spambots accounted for 36.4%. 1,304 (12.6%) tweets shared health information. 80.6% of the information-sharing tweets were medically correct. The percentage of information-sharing tweets significantly rose during the campaign (77.1% to 81.1%). |
| Salako (2017) | 217,395 impressions | Feb-Nov 2015 | Exposure, high-level engagement | 217,395 impressions on Twitter and 67,923 on Facebook. Views increased steadily throughout the campaign and returned to baseline after the campaign. Approximately 9000 participants were present at the Guinness World Record attempt for the largest human ribbon formed. |
| Thackeray (2013) | 1,351,823 tweets | September - November 2012. | Nature of information, exposure, medium-level engagement | Users represented: individuals (93%), organizations (7%), and celebrities (0.3%). Tweets were mostly about wearing pink and promoting walks/runs and fundraising. 797,827 users tweeted. Tweets from celebrities were more likely to be retweeted. Tweets spiked in the first few days, with a peak of 125,278 on October 1^st^. |
| Theiss (2016) | 574 Facebook posts | August 2014 - April 2016 | low-level and medium-level engagement | Posts that included a photo had the highest engagement rate (ER=5.6). Posts shared in 2014 had the highest engagement rate 6.9, whereas those posted in 2016 had the lowest ER (3.8). |
| Vos (2019) | 1.2 million Tweets | September-October 2016 | Nature of information, medium-level engagement | Most messages were related to prostate cancer (n=16,890, 81%). Stiller’s tweet received 3551 retweets. Retweeting resulted in ~21.2 million additional exposures. There was an increase in prostate cancer messages on the day of Stiller’s announcement, which decreased by 10% a day returning to preannouncement levels 2 days later. |
| Vraga (2018) | 5,444,470 Tweets | January 2015- February 2016 | Medium-level engagement | There were 2,075,168 mentions of breast cancer during October (BCAM), compared with 400,680 tweets about Movember and 65,820 tweets mentioning prostate cancer during November. Breast cancer received more mentions on Twitter in November (284,015 posts) than prostate cancer. Both campaigns started strong at the beginning of the corresponding month and tapered off. |
| Xu (2016) | 19,818,236 Tweets | April 2014 - January 2015 | Reach, medium-level engagement | Campaigns mainly reached White users (93%) compared to African American (7%), Asian or Hispanic (0.6%) users. Colorectal cancer received least attention. White users mentioned breast and prostate cancer more than other ethnicities. The frequency of cancer tweets was highest during campaigns then decreased to levels lower than seen leading up to campaigns. |
| Qualitative research designs | | | | |
| Abramson (2015) | 584 posts analyzed plus 2500 responses | October 2010 | Nature of information | Themes included open mic communication, scarcity of health information, the commodification of breast cancer, unpredictable locations of conversation, and the use of gendered images and language. |
| Bravo (2016a) | 2400 Twitter conversations | November 2013 | Nature of information | The major themes identified in the tweets were fundraising as a priority (34%), making a change to men’s health (18%), the campaign as a moustache contest rather than a charity (26%), the use of masculine metaphors/imagery (9%), and the role of women as moustache supporters (4%). |
| Mixed methods research designs | | | | |
| Good Things Foundation (2018) | Females, 50-70 years old in North Midlands, UK | 2014-2018 | Nature of information, high-level engagement | Comments showed the service allowed for better communication and was easy to use. Using Facebook to deliver information and book breast screening appointments improved attendance at screening by an average of 12.9% across 7 sites. |

1. Outcomes measured were adapted from Neiger et al key performance indicators and metrics related to social media use in health promotion (insights, exposure, reach and engagement low/medium/high) and nature of information delivered and shared. [↑](#footnote-ref-1)
2. Key-word targeted videos are videos that appear if specific keywords are used. Pre-roll videos are videos played before a selected video. [↑](#footnote-ref-2)
